# Periodontal disease as an indicator of chronic non-communicable diseases: evidence from literatures

**DOI:** 10.1186/1471-2458-12-S2-A26

**Published:** 2012-11-27

**Authors:** Tuti Ningseh Mohd Dom, Shahida Mohd Said, Aznida Firzah Abdul Aziz, Mohd Rizal Abdul Manaf, Syed Mohamed Aljunid

**Affiliations:** 1 United Nations University International Institute for Global Health, Jalan Yaacob Latiff, 56000 Kuala Lumpur, Malaysia; 2 Department of Dental Public Health, Faculty of Dentistry, Universiti Kebangsaan Malaysia, Jalan Raja Muda Abdul Aziz, 50300 Kuala Lumpur, Malaysia; 3 Department of Periodontology, Faculty of Dentistry, Universiti Kebangsaan Malaysia, Jalan Raja Muda Abdul Aziz, 50300 Kuala Lumpur, Malaysia; 4 Department of Community Health, Universiti Kebangsaan Malaysia Medical Centre, Jalan Yaacob Latiff, 56000 Kuala Lumpur, Malaysia

## Background

Periodontal disease has been recognised as a major global public health problem because of its prevalence, economic impact and health consequences. Nevertheless, its burden to the health care system is often largely neglected. Identification of non-communicable diseases which are associated with periodontal disease and understanding of the strengths of the proposed associations is important so that health care providers may identify common risk factors and work together towards attaining synergistic control of these diseases. This paper reviews recent data on association between periodontal disease and non-communicable diseases.

## Method

Electronic literature search was done using Medline database for period between 2001 to March 2012. The search was limited to clinical/ human studies published in English and included the following levels of available evidence: systematic reviews, narrative reviews, clinical trials as well as cross-sectional and cohort studies. Selected papers were articles related to studies investigating whether or not a disease is a risk factor for periodontal disease. Integration of oral and periodontal health promotion into management of these systemic diseases in primary care settings were also appraised.

## Results

An overwhelming body of evidence supports the association between periodontal disease and non-communicable diseases such as cardiovascular disease (OR 1.1 - 2.4), type 2 diabetes mellitus (OR 1.5 - 2.3) and chronic respiratory diseases (1.1 - 2.0). Etiological and pathological links between periodontal disease and these diseases have also been suggested. There is global support towards control of periodontal disease through common risk factor approaches with these systemic conditions.

## Conclusions

Findings support associations between periodontal disease and some important non-communicable diseases. A focus on control of periodontal disease in overall primary health care will potentially reduce the rate of cardiovascular diseases, diabetes and chronic respiratory diseases.

